# NHS-Galleri trial: Enriched enrolment approaches and sociodemographic characteristics of enrolled participants

**DOI:** 10.1177/17407745241302477

**Published:** 2025-01-25

**Authors:** Charles Swanton, Velicia Bachtiar, Chris Mathews, Adam R Brentnall, Ian Lowenhoff, Jo Waller, Martine Bomb, Sean McPhail, Heather Pinches, Rebecca Smittenaar, Sara Hiom, Richard D Neal, Peter Sasieni

**Affiliations:** 1 University College London, London, UK; 2 GRAIL Bio UK Ltd., GRAIL, Inc., London, UK; 3 King’s College London, London, UK; 4 Queen Mary University of London, London, UK; 5 NHS England, London, UK; 6 University of Exeter, Exeter, UK

**Keywords:** Multi-cancer early detection, liquid biopsy, cell-free nucleic acids, population screening, randomised controlled trial, cancer, equity, socioeconomic status, ethnicity, clinical trial recruitment

## Abstract

**Background/aims:**

Certain sociodemographic groups are routinely underrepresented in clinical trials, limiting generalisability. Here, we describe the extent to which enriched enrolment approaches yielded a diverse trial population enriched for older age in a randomised controlled trial of a blood-based multi-cancer early detection test (NCT05611632).

**Methods:**

Participants aged 50–77 years were recruited from eight Cancer Alliance regions in England. Most were identified and invited from centralised health service lists; a dynamic invitation algorithm was used to target those in older and more deprived groups. Others were invited by their general practice surgery (GP-based Participant Identification Centres in selected regions); towards the end of recruitment, specifically Asian and Black individuals were invited via this route, as part of a concerted effort to encourage enrolment among these individuals. Some participants self-referred, often following engagement activities involving community organisations. Enrolment took place in 11 mobile clinics at 151 locations that were generally more socioeconomically deprived and ethnically diverse than the England average. We reduced logistical barriers to trial participation by offering language interpretation and translation and disabled access measures. After enrolment, we examined (1) sociodemographic distribution of participants versus England and Cancer Alliance populations, and (2) number needed to invite (NNI; the number of invitations sent to enrol one participant) by age, sex, index of multiple deprivation (IMD) and ethnicity, and GP surgery-level bowel screening participation.

**Results:**

Approximately 1.5 million individuals were invited and 142,924 enrolled (98% via centralised health service lists/invitation algorithm) in 10.5 months. The enrolled population was older and more deprived than the England population aged 50–77 years (73.3% vs 56.8% aged 60–77 years; 42.3% vs 35.3% in IMD groups 1–2). Ethnic diversity was lower in the trial than the England population (1.4% vs 2.8% Black; 3.3% vs 5.3% Asian). NNI was highest in Black (32.8), Asian (28.2) and most-deprived (21.5) groups, and lowest in mixed ethnicity (8.1) and least-deprived (4.6) groups.

**Conclusions:**

Enrolment approaches used in the NHS-Galleri trial enabled recruitment of an older, socioeconomically diverse participant population relatively rapidly. Compared with the England and Cancer Alliance populations, the enrolled population was enriched for those in older age and more deprived groups. Better ethnicity data availability in central health service records could enable better invitation targeting to further enhance ethnically diverse recruitment. Future research should evaluate approaches used to facilitate recruitment from underrepresented groups in clinical trials.

## Introduction

Cancer is the leading cause of death in England and most other high-income countries, especially among older adults.^1^ Equitable access to early cancer detection is needed to reduce cancer burden in the general population and reduce disparities in cancer burden between population subgroups by socioeconomic deprivation and ethnicity.

In England, those living in more socioeconomically deprived neighbourhoods have lower screening participation, higher incidence of late-stage cancer and cancer mortality, and shorter life expectancy than those living in less deprived neighbourhoods.^2–6^ Cancer stage at diagnosis and mortality differ between ethnic groups in England, though the pattern varies by cancer type.^7–10^ In addition, populations in areas with higher deprivation are likely to be more ethnically diverse; the intersection of subpopulations likely has complex negative impacts on cancer outcomes in these populations.^11,12^

Given the sociodemographic variation in cancer burden, inclusive recruitment of a diverse clinical trial population is required to enable an analysis that is generalisable to the intended-use population.^13,14^ Despite published guidance on clinical trial inclusivity (e.g. NIHR INCLUDE and Trial Forge),^14,15^ those experiencing greater socioeconomic deprivation, those from minority ethnic groups, and older individuals are often underrepresented in clinical trials.^16–19^

The clinical utility of a previously validated^20^ blood-based multi-cancer early detection test is currently being assessed among adults aged 50–79 years in England, in the large, pragmatic, randomised controlled NHS-Galleri trial (NCT05611632).^21^ This multi-cancer early detection test measures the extent and nature of cell-free DNA methylation. It has been shown to detect a shared cancer signal across more than 50 different cancer types from a single blood sample and predict a cancer signal origin (i.e. the tissue of origin) with 88.0% accuracy.^22^

In this paper, we aim to (1) describe the various dynamic, enriched enrolment approaches used in the NHS-Galleri trial; (2) assess the extent to which these approaches enabled the recruitment of a sociodemographically diverse sample enriched for older age compared with the populations from which participants were recruited; and (3) describe invitation and enrolment patterns and determine how many invitations were sent to enrol one participant (the NNI) by various sociodemographic factors.

## Methods

### Trial setting and recruitment targets

Recruitment was between 31 August 2021 and 16 July 2022, lasting 46 weeks (Supplementary Material, Figure S1). A national-level communications campaign, including trial coverage in national press, radio, and television outlets, was coordinated to coincide with recruitment opening.

We aimed to recruit individuals aged 50–77 years across sexes and major ethnic groups in England,^23^ with proportionally more individuals invited from older age groups and more deprived neighbourhoods. The trial team selected Cancer Alliances (area-based NHS organisational units that coordinate cancer care and aim to improve outcomes for patients locally) based on their higher cancer mortality, worse early-stage diagnosis, and greater ethnic diversity (Supplemental Material).^21^ Eligible individuals excluded those who had opted out from having their confidential patient information used for research purposes via the national data opt-out service, or from the NHS-Galleri trial via NHS DigiTrials or the trial website.^21^

### Recruitment routes and enriched enrolment approaches

#### Targeted invitation from algorithm-generated lists via NHS DigiTrials

Most participants were recruited via the NHS DigiTrials service,^24^ which sent invitations to stratified random samples from a pool of eligible, general practice (GP)-registered individuals from centralised health service lists across the eight participating Cancer Alliances.^21^ NHS DigiTrials held information on invitee age, sex and GP surgery at the time of invitation.

The Cancer Prevention Trials Unit at King’s College London used an algorithm^25^ to generate invitation lists following weekly reviews of enrolments. Using central patient registration data, GP surgeries serving individuals in the eligible pool were ranked and prioritised for inclusion by distance from their nearest trial location, Index of Multiple Deprivation (IMD, an area-based measure of relative deprivation^26^) and (from week 35) ethnic distribution. Groups of individuals registered at each GP surgery could be included or excluded from invitation lists based on age and sex. A statistical model to predict enrolment based on age, sex, proxy IMD (derived from GP postcodes mapped to small geographic areas, known as lower-layer super output areas) and GP surgery-level bowel screening participation also fed into the algorithm. The algorithm adjusted the invitation distributions based on what was needed to meet recruitment targets.^25^ Bowel screening participation was a positive indicator of invitation uptake; to offset this, more invitations were sent to those registered at GP surgeries with lower bowel screening participation.^25^ These adjustments aimed to avoid under-representation of groups less likely to participate in screening,^25^ underpowering due to inadequate cancer event rates^27^ and to compensate for healthy volunteer bias.^27^

Researchers at King’s College London held names and addresses of those who accepted invitations in order to schedule appointments. Only de-identified data were passed to GRAIL, Inc. (Menlo Park, CA; the sponsor of the NHS-Galleri trial^21^) for subsequent analysis.

#### GP participant identification centre invitation

Other participants were recruited via direct invitation from GP surgery-based Participant Identification Centres (PICs). PICs are NHS organisations that can use practice records to invite potential trial participants. GP PICs are not trial research sites and are external to and separate from the organisations running this trial.

There were two rounds of recruitment via GP PICs. The first round took place in the first 2 weeks of the trial and was used to test whether functional elements were operating correctly after launch. This round took place at sites in Cheshire and Merseyside and South East London (the first two mobile clinics to open in the trial), and collectively sent out 8407 invitations to eligible individuals. The second round took place in weeks 38–40 (towards the end of recruitment) at sites in South East London and East Midlands, selected for their greater ethnic diversity; these sites sent 5789 and 1567 invitations to eligible individuals whose ethnicities were coded in GP records as Black or Asian, respectively.

#### Open enrolment

Eligible individuals who were not invited via NHS DigiTrials from algorithm-generated lists, or by GP PICs, could self-refer (‘open enrolment’), often following engagement activities involving voluntary and community sector organisations. We worked with local champions (e.g. community groups, health organisations and faith leaders) to distribute information on the trial and how to enrol, and arranged coverage of the trial by local radio and television stations. We developed a social media toolkit to guide communications, information leaflets for distribution and (e-) posters to be displayed in community hubs and at local events.

### Reducing barriers to participation

All trial appointments were conducted in mobile clinics generally stationed in socioeconomically deprived neighbourhoods with good public transport within the eight Cancer Alliances. The trial team identified these locations using the online data mapping and analysis tool SHAPE^28^ in collaboration with local health teams. Seven large mobile clinics (each with capacity for conducting blood draws for up to eight individuals simultaneously) were stationed in car parks with high general public footfall (e.g. retail parks, supermarkets, leisure centres and sports stadiums). Four small mobile clinics (each with capacity for conducting blood draws for up to two individuals simultaneously) were used where access and parking was limited (e.g. car parks in rural locations, places of worship, health centres and parks).

We provided translated participant information sheets (in Bengali, Urdu, Gujarati and Punjabi) and on-demand language interpretation at registration and during appointments. We offered wheelchair and step-free access and hearing assistance, including sign language interpretation, to all individuals (Supplemental Material).

Invitations, information sheets, and consent forms were developed before the start of the trial with input from behavioural science researchers at King’s College London and a sociodemographically diverse group of the public (Supplemental Material).

### Diversity and inclusion in the NHS-Galleri trial

To gauge the extent to which a sociodemographically diverse trial participant population was enrolled, we compared the demographic and health-associated characteristics of those enrolled with national statistics in England and the Cancer Alliances where recruitment took place.

For trial participants, data were from the baseline questionnaire filled out at enrolment. This included self-reported age, sex, actual IMD (derived from participant postcodes mapped to lower-layer super output areas), ethnicity, body mass index (BMI) calculated from self-reported weight and height, smoker status and alcohol drinker status. We used Office for National Statistics and Health Survey for England data for comparison (Supplemental Material).

### Impact of enriched enrolment approaches on enrolment of participants by ethnicity

Enrolment approaches intended to boost ethnic diversity were: stationing mobile clinics in more ethnically diverse and socioeconomically deprived areas (weeks 37–45), inviting Asian and Black individuals via GP PIC sites (weeks 38–40), engagement activities involving voluntary and community sector organisations (weeks 36–45), and adjusting algorithm-generated invitation lists by proxy ethnicity (week 35 onwards). A descriptive analysis of the collective impact of these approaches on enrolment is shown in Figure S2 in the Supplemental Material.

### Enrolment by sociodemographic characteristics among those invited via NHS DigiTrials from algorithm-generated lists

We estimated NNI of those invited via NHS DigiTrials from algorithm-generated lists by age, sex, IMD, ethnicity and GP surgery-level bowel screening participation. For invitees, NHS DigiTrials provided age, sex and registered GP surgery, and we derived proxy values for IMD, ethnicity, and bowel screening participation. We used the 2019 English indices of deprivation^26^ to assign an IMD to each GP surgery based on the lower-layer super output area in which it was located. IMD is based on indicators from seven domains including income, employment and education. We applied GP surgery-level patient ethnic distributions (derived from GP surgery lower-layer super output areas) to the invited population, assuming equal age and sex distributions within each ethnic group. We based bowel cancer screening participation on percent bowel cancer screening participation at each participant’s registered GP surgery, according to 2021–2022 Fingertips public health data.^29^

We also compared the proportion of invitations and enrolments in each ethnic group by Cancer Alliance to determine whether invitation and enrolment of Asian and Black individuals were specifically enriched in East Midlands and South East London. These analyses were descriptive.

### Ethics approval and informed consent

The trial was conducted according to the guidelines of the Declaration of Helsinki and received ethical approval from Wales Research Ethics Committee 1 (Ref: 21/WA/0141). The trial also received Health Research Authority approval with support from the Confidentiality Advisory Group (Ref:21/CAG/0056), under Regulation 5 of the Health Service Regulations 2002 (‘Section 251 support’), for NHS Digital (now merged into NHS England) to send out invitation letters to eligible invitees to seek consent. All participants provided written informed consent before participation in the trial.

## Results

**Figure 1.**
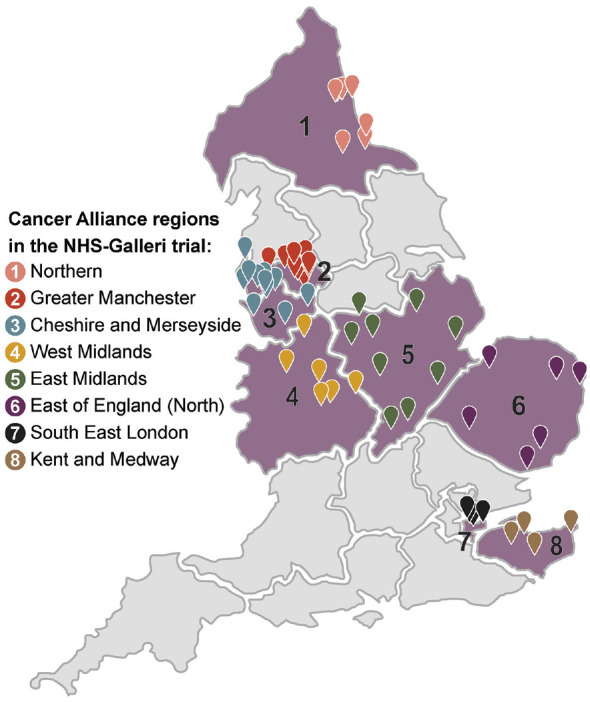
Map of geographical locations of the mobile clinics at which enrolment appointments were held.

Overall, 1,496,311 individuals were invited (1,480,548 via NHS DigiTrials) and 142,924 participants were enrolled in the NHS-Galleri trial over 10.5 months. All enrolment appointments, which included blood draws, were conducted in 11 mobile units at 151 locations (Figure 1).

### Demographic and health-associated characteristics of the NHS-Galleri trial enrolled participant population

By design, the NHS-Galleri trial enrolled population was enriched for older age groups, with proportionally more participants aged 60–64, 65–69, 70–74, and 75–77 years than that of England or the eight Cancer Alliances (Table 1). The median age of the NHS-Galleri trial enrolled population was 66 years (interquartile range, 59–71). There were similar numbers of female and male individuals in the enrolled population, also by design. Compared with the Cancer Alliance and England populations aged ≥50 years, the enrolled population included proportionally more individuals in the White and Mixed ethnic groups and proportionally fewer in the Asian, Black and Other ethnic groups. The trial recruited proportionally more individuals in the two most-deprived IMD quintiles and fewer in the two least-deprived quintiles compared with the England population; this pattern was present but much less pronounced between the enrolled and Cancer Alliance populations.

**Table 1.** Demographic characteristics of the participants enrolled in the NHS-Galleri trial.

		NHS-Galleri trial	Cancer alliance regions^a^	England
Years of Age^b^	50–54	15,436 (10.8)	1,748,000 (21.5)	3,908,000 (21.9)
	55–59	22,460 (15.7)	1,723,000 (21.2)	3,806,000 (21.3)
	60–64	26,815 (18.8)	1,488,000 (18.3)	3,256,000 (18.2)
	65–69	30,813 (21.6)	1,279,000 (15.7)	2,767,000 (15.5)
	70–74	30,428 (21.3)	1,297,000 (15.9)	2,797,000 (15.7)
	75–77	16,631 (11.6)	613,000 (7.5)	1,323,000 (7.4)
Sex^b^	Female	71,848 (50.3)	4,176,000 (51.3)	9,169,000 (51.4)
	Male	71,076 (49.7)	3,972,000 (48.7)	8,687,000 (48.7)
Ethnicity^c^	White	133,171 (93.2)	8,952,000 (91.8)	19,206,000 (89.9)
	Asian	4725 (3.3)	415,000 (4.3)	1,127,000 (5.3)
	Black	2056 (1.4)	236,000 (2.4)	594,000 (2.8)
	Other	627 (0.4)	80,000 (0.8)	267,000 (1.3)
	Mixed	1544 (1.1)	69,000 (0.7)	177,000 (0.8)
IMD Quintile^b,d^	1 – Most Deprived	32,347 (22.7)	1,744,000 (21.4)	2,948,000 (16.7)
	2	28,067 (19.6)	1,562,000 (19.2)	3,285,000 (18.6)
	3	30,125 (21.1)	1,661,000 (20.4)	3,663,000 (20.8)
	4	28,963 (20.3)	1,655,000 (20.3)	3,837,000 (21.8)
	5 – Least Deprived	22,953 (16.0)	1,527,000 (18.7)	3,894,000 (22.1)

Data are presented as n (%). Percentages may not sum to 100% across groups due to rounding error.

a Northern, Cheshire and Merseyside, Greater Manchester, West Midlands, East Midlands, East of England (North), South East London, and Kent and Medway.

b Age range 50–77 years.

c Age range ≥50 years.

d Based on LSOAs in England.

IMD: index of multiple deprivation; LSOA: lower-layer super output area.

The prevalence of self-reported current smokers enrolled in the NHS-Galleri trial (6.6%) was towards the low end of the range among those aged ≥45 years in the England population (4%–13%), according to the 2019 NHS Health Survey for England (Table 2). This pattern was similar for current drinkers (78.5% in the trial vs 77%–85% in the England population).

**Table 2.** Health-associated characteristics of the participants enrolled in the NHS-Galleri trial.

		NHS-Galleri trial (N = 142,924)		England^a^			
		% (n)	Median age, years (IQR)	Aged 45–54 years	Aged 55–64 years	Aged 65–74 years	Aged 75 years or older
Smoker Status^b^	Current Smoker	6.6 (9455)	62 (56–68)	12	13	8	4
	Former Smoker	38.1 (54,443)	67 (60–72)	23	26	35	39
	Non-Smoker	54.7 (78,220)	65 (59–71)	65	61	57	57
Alcohol Drinker Status^b^	Current Drinker	78.5 (112,257)	65 (59–71)	81	85	85	77
	Former Drinker	15.2 (21,653)	66 (59–72)	19	15	15	23
	Non-Drinker	5.6 (8068)	65 (58–71)				
BMI (kg/m^2^)	<18.5	0.9 (1284)	68 (61–73)	<1	1	2	1
	18.5–24.9	31.5 (44,991)	66 (60–72)	27	27	23	28
	25.0–29.9	39.1 (55,953)	66 (59–72)	40	39	39	45
	≥30.0	27.6 (39,467)	64 (58–70)	36	38	39	28

England data were available only for adults aged 45–54, 55–64, 65–74, and 75 years or older. Data for the participants enrolled in the NHS-Galleri trial are presented as % unless otherwise specified. Percentages may not sum to 100% across groups due to rounding error.

a Absolute numbers not available; percentages available only to the nearest integer.

b The definitions of smoker and alcohol drinker categories differed between the NHS-Galleri trial participant survey and the Health Survey for England (Supplemental Material).

BMI: body mass index; IQR: interquartile range.

Proportionally, there were slightly more individuals with a BMI of 18.5–24.9 kg/m^2^ enrolled in the trial (31.5%) than the England population aged ≥45 years (23%–28%). The proportions of enrolled individuals in other BMI groups were within the ranges observed in the England population (Table 2).

### Enrolment by route

Overall, 139,601 (97.7%) participants were enrolled via NHS DigiTrials from algorithm-generated invitation lists, 2358 (1.6%) via open enrolment and 965 (0.7%) via GP PICs.

There were proportionally more Asian and Black individuals recruited via GP PICs and open enrolment compared with those recruited via NHS DigiTrials (Table 3), though absolute numbers were much smaller. Of those enrolled via GP PICs, 167 (17.3%) were enrolled from the targeted ethnicity drive in 2022, of which 58.1% were Black, 22.2% were Asian and 14.4% were Mixed ethnicity. Ethnicities of the remaining 5.3% were discordant with GP PIC records.

**Table 3.** Demographic characteristics of the enrolled participants in the NHS-Galleri trial by recruitment route.

		NHS DigiTrials (from algorithm-generated invitation lists) N = 139,601	Open enrolment N = 2358	GP PIC sites N = 965	All routes N = 142,924
Years of Age	50–54	14,850 (10.6)	429 (18.2)	157 (16.3)	15,436 (10.8)
	55–59	21,859 (15.7)	470 (19.9)	131 (13.6)	22,460 (15.7)
	60–64	26,081 (18.7)	506 (21.5)	228 (23.6)	26,815 (18.8)
	65–69	30,140 (21.6)	434 (18.4)	239 (24.8)	30,813 (21.6)
	70–74	29,909 (21.4)	358 (15.2)	161 (16.7)	30,428 (21.3)
	75–77	16,426 (11.8)	157 (6.7)	48 (5.0)	16,631 (11.6)
Sex	Female	69,868 (50.0)	1444 (61.2)	536 (55.5)	71,848 (50.3)
	Male	69,733 (50.0)	914 (38.8)	429 (44.5)	71,076 (49.7)
Ethnicity	White	130,386 (93.4)	2055 (87.2)	730 (75.6)	133,171 (93.2)
	Asian	4508 (3.2)	163 (6.9)	54 (5.6)	4725 (3.3)
	Black	1855 (1.3)	76 (3.2)	125 (13.0)	2056 (1.4)
	Other	604 (0.4)	13 (0.6)	10 (1.0)	627 (0.4)
	Mixed	1466 (1.1)	43 (1.8)	35 (3.6)	1544 (1.1)
IMD Quintile	1 – Most Deprived	31,760 (22.7)	365 (15.4)	222 (23.0)	32,347 (22.7)
	2	27,312 (19.6)	456 (19.3)	299 (31.0)	28,067 (19.6)
	3	29,339 (21.0)	529 (22.5)	257 (26.6)	30,125 (21.1)
	4	28,254 (20.2)	539 (22.9)	170 (17.6)	28,963 (20.3)
	5 – Least Deprived	22,478 (16.1)	461 (19.5)	14 (1.4)	22,953 (16.0)

All data are presented as n (%). Percentages may not sum to 100% across groups due to rounding error.

IMD: index of multiple deprivation; GP PIC: general practice participant identification centre.

Invitations were targeted by GP surgery-level proxy ethnicity from week 35 of recruitment, but the percentages of ethnic minority groups enrolled via NHS DigiTrials remained lower than in the England and Cancer Alliance populations (Table 3).

### Enrolment by ethnicity

Overall, ∼60% of enrolment from the Asian and Black ethnic groups and ∼45% of enrolment from Mixed and Other ethnic groups took place later in recruitment (weeks 35–45), compared with ∼30% of enrolment from the White ethnic group (Supplemental Material, Figure S2). This corresponded to the period in which the following approaches were implemented: adjusting algorithm-generated lists by GP surgery-level ethnic distribution; stationing mobile clinics in more deprived and diverse areas; engagement activities involving voluntary and community sector organisations; and GP PIC recruitment of those from minority ethnic groups.

### Invitations, enrolments and NNI by sociodemographic group

For those enrolled via NHS DigiTrials from algorithm-generated invitation lists, the NNI was 10.6 overall. The NNI was higher for younger versus older age groups, male versus female participants, Asian and Black versus White and Mixed ethnic groups, and more deprived groups (Table 4). The NNI was higher for GP surgeries with lower bowel screening participation.

**Table 4.** Number of individuals enrolled and invited via NHS DigiTrials from algorithm-generated lists, and the number of invitations needed to obtain one enrolment (number needed to invite, NNI) by demographic characteristics and GP surgery-level bowel screening participation.

		Enrolled	Invited^a^	NNI (95% CI)
Years of Age	50–54	14,850	219,316	14.8 (14.58–14.96)
	55–59	21,859	267,932	12.3 (12.13–12.39)
	60–64	26,081	265,017	10.2 (10.06–10.26)
	65–69	30,140	269,428	8.9 (8.86–9.02)
	70–74	29,909	280,408	9.4 (9.29–9.46)
	75–77	16,426	174,950	10.7 (10.52–10.78)
Sex	Female	69,868	701,935	10.0 (9.99–10.11)
	Male	69,733	775,116	11.1 (11.05–11.18)
Ethnicity	White	130,386	1,268,180	9.7 (9.68–9.77)
	Asian	4508	127,088	28.2 (27.52–28.88)
	Black	1855	60,911	32.8 (31.63–34.10)
	Other	604	8,941	14.8 (13.88–15.79)
	Mixed	1466	11,863	8.1 (7.77–8.43)
IMD Quintile^b^	1 – Most Deprived	31,760	683,924	21.5 (21.34–21.73)
	2	27,312	330,603	12.1 (11.99–12.22)
	3	29,339	215,428	7.3 (7.28–7.41)
	4	28,254	151,761	5.4 (5.32–5.42)
	5 – Least Deprived	22,478	103,235	4.6 (4.55–4.64)
Bowel Screening Participation Quintile^c^	1 – Low Participation	21,172	473,850	22.4 (22.14–22.63)
	2	39,175	426,666	10.9 (10.81–10.98)
	3	41,595	349,584	8.4 (8.34–8.47)
	4	18,204	145,086	8.0 (7.88–8.06)
	5 – High Participation	12,600	68,478	5.4 (5.36–5.51)

a Based on GP surgery-level proxy values for IMD, ethnicity, and bowel screening participation.

b Based on LSOAs in England.

c Based on quintiles of screening participation for GP surgeries in England according to percentage participation as follows: Q1, <56.0%; Q2, 56.0%–62.9%; Q3, 63.0%–66.9%; Q4, 67.0%–70.9%; Q5, >71.0%.

95% CI: 95% confidence interval; GP: general practice; IMD: index of multiple deprivation; LSOA: lower-layer super output area.

Proportionally more Asian individuals were invited and enrolled in East Midlands compared with the ethnic distribution in this Cancer Alliance (Table 5). Proportionally more Black individuals were invited in South East London compared with the ethnic distribution in this Cancer Alliance, but this did not translate to proportionally greater enrolment relative to the overall population.

**Table 5.** Number of individuals enrolled and invited via NHS DigiTrials from algorithm-generated lists in East Midlands, South East London and the other six Cancer Alliances combined.

		Population	Invited^a^	Enrolled
East Midlands	White	1,686,410 (92.3)	142,663 (76.4)	16,453 (88.9)
	Asian	91,318 (5.0)	35,713 (19.1)	1479 (8.0)
	Black	27,945 (1.5)	5535 (3.0)	241 (1.3)
	Other	10,930 (0.6)	1272 (0.7)	75 (0.4)
	Mixed	10,536 (0.6)	1503 (0.8)	181 (1.0)
	Missing	N/A	N/A	77 (0.4)
	Total	1,827,139 (100.0)	186,686 (100.0)	18,506 (100.0)
South East London	White	355,939 (68.5)	71,685 (63.5)	6562 (79.9)
	Asian	36,413 (7.0)	7813 (6.9)	345 (4.2)
	Black	96,312 (18.5)	28,339 (25.1)	730 (8.9)
	Other	18,750 (3.6)	2139 (1.9)	138 (1.7)
	Mixed	12,258 (2.4)	2907 (2.6)	345 (4.2)
	Missing	N/A	N/A	92 (1.1)
	Total	519,672 (100.0)	112,883 (100.0)	8212 (100.0)
Other Cancer Alliances Combined^b^	White	6,909,675 (93.3)	1,053,832 (89.5)	107,371 (95.1)
	Asian	287,599 (3.9)	83,562 (7.1)	2684 (2.4)
	Black	111,691 (1.5)	27,037 (2.3)	884 (0.8)
	Other	50,505 (0.7)	5530 (0.5)	391 (0.3)
	Mixed	46,225 (0.6)	7453 (0.6)	940 (0.8)
	Missing	N/A	N/A	613 (0.5)
	Total	7,405,695 (100.0)	1,177,414 (100.0)	112,883 (100.0)

All values are presented as n (%). Percentages may not sum to 100% across groups due to rounding error.

^a^Based on GP surgery-level proxy values.

^b^Cheshire and Merseyside, East of England (North), Greater Manchester, Kent and Medway, Northern, and West Midlands.

GP: general practice; N/A: not available.

## Discussion

The enrolment approaches used in the NHS-Galleri trial enabled the recruitment of a large, sociodemographically diverse population of 142,924 individuals exceptionally rapidly (10.5 months). By design, we enrolled proportionally more participants in older age groups than the England population and that of the Cancer Alliances from which participants were recruited. We also enrolled proportionally more participants in more deprived groups than both the England and Cancer Alliance populations. The invitation algorithm enabled us to meet enrolment targets by age, sex and deprivation group, which contributes to sufficient powering to meet the trial objectives.^25^

Enriched enrolment of Asian participants was observed in East Midlands. This was likely due to high geographic clustering of the Asian population^30^ in this Cancer Alliance, along with the prioritisation of GP surgeries for inclusion in algorithm-generated lists by ethnicity from week 35. It was not possible to target invitations via the algorithm based on person-level ethnicity as these data were not held in the centralised health service lists. Self-reported ethnicity was only available for participants after enrolment, once they had filled in the baseline questionnaire, making it difficult to track enrolment by ethnicity in real time. We successfully targeted invitations by person-level ethnicity during the second round of recruitment via GP PICs, but the percentage enrolled during this round was small (0.1% overall). In addition, ethnicity coding was found to be highly variable between GP PIC sites during feasibility searches prior to initiating recruitment. More accurate and consistent coding of ethnicity data in GP records is required to achieve more effective targeting by ethnicity.

The proportion of the trial population recruited via open enrolment was small (1.6%) but was enriched for Asian and Black individuals compared with the England and Cancer Alliance populations. Engagement activities involving community and volunteer organisations and stationing mobile clinics in more ethnically diverse communities may have contributed to this. We did not specifically assess their impact in this study, though previous reports indicate that such approaches may be effective as part of a holistic enrolment strategy.^31^

Enrolment targets in the trial were set using 2011 census data, which were the most recently available at the time; however, ethnic diversity increased between 2011 and 2021. The proportions of those in ethnic minority groups in our study was more similar to the 2011 census data than the 2021 census data for both the England and Cancer Alliance populations (Supplemental Material, Table S1).

Step-free access, hearing assistance, and language translation services supported 6.1% of appointments (Supplemental Material). These numbers represent individuals who may not have otherwise participated in the trial. Although physical and some communication barriers were minimised, other barriers (e.g. greater logistical difficulty travelling to appointments, unconscious ableism)^32^ may have remained. Future research using qualitative methods could provide insights into the effectiveness of accessibility measures from recruitment staff and participant perspectives.

The NNI among those invited via NHS DigiTrials was higher for those in the youngest vs oldest age groups. The reasons for this are currently unknown; most literature on trial participation by age focuses on the phenomenon of lower participation among those aged ≥65 years compared with <65 years.^18^

Deterrents to participation among more deprived and ethnic minority groups, for whom the NNI was also higher, include greater mistrust in clinical trials and the healthcare system, time commitments, costs, transportation issues, lower comprehension of cancer and clinical trials, and lower literacy/health literacy levels.^31,33,34^ Patient and public involvement focus groups conducted prior to the NHS-Galleri trial to inform the development of our communications materials (Supplemental Material) found that among Asian and Black individuals, barriers to participation included mistrust in private organisations running clinical trials, anticipated stress over diagnostic waiting times after a positive result, and concerns about the use of blood samples for purposes unrelated to the trial. Although efforts were made to mitigate the impact of these concerns, particularly in our trial communications, they may still have affected enrolment. In addition, NNI values were not adjusted, therefore differences between groups may be partially due to other sociodemographic factors, and the intersectional effects of these factors. Future research should focus on behavioural drivers and barriers to participation, and their interaction with demographic characteristics, to improve enrolment among those less likely to participate.

To compensate for health inequalities associated with low cancer screening uptake, we invited more individuals registered at GP surgeries with lower bowel screening participation, who had a higher NNI. Our results demonstrate that bowel screening data can be used to support recruitment of those less likely to participate, who are thus likely to be underrepresented in screening trials.

The economic, logistic and psychosocial factors affecting participation in national screening programmes, including low perceived personal relevance of screening and low trust in healthcare providers,^35,36^ may have affected participation in the NHS-Galleri trial. A recent systematic review indicates that >50% of individuals with cancer who are invited to participate in treatment clinical trials agree to do so,^37^ but there is currently no equivalent estimate for screening trials. Our study therefore provides valuable benchmarking information for enrolment rates.

This is the first time that NHS DigiTrials has been used in a non-COVID-19 study. It is therefore a major precedent for other large-scale, population-based trials. The use of NHS DigiTrials to identify and invite eligible participants in our study, along with national media campaigns and local communications activities, enabled exceptionally rapid recruitment in the NHS-Galleri trial (10.5 months) compared with other screening trials of a similar scale (3–6 years).^38–41^

Other national-level clinical trials on a similar scale have reported difficulties enrolling a sufficiently ethnically diverse population, despite a concerted effort to target ethnic minority groups.^38,42^ Recently, some trials (BLUE-C bowel screening trial [NCT04144738]) have reported success in this arena.^43^ However, screening trials often lack specific reporting on strategies to improve diversity and inclusion.^39–41,44,45^ This highlights a need for both improved trial design and better reporting of enrolment approaches and diversity, which we have attempted to address in this article.

This study has some limitations. Because it was not designed to evaluate the impact of individual approaches on enrolment, we could not say which approaches were most effective for enrolling underrepresented groups. This could potentially be evaluated in future by collecting information on how individuals in underrepresented groups heard about the trial and were recruited, and reasons for non-participation among those in underrepresented groups who decided not to participate. The accuracy of proxy ethnicity and bowel screening participation also could not be determined, which may have affected the accuracy of the NNI values reported here.

## Conclusion

Enrolment approaches used in the NHS-Galleri trial enabled the rapid recruitment of an older, socioeconomically diverse participant population. Compared with the England and Cancer Alliance populations, the enrolled population was enriched for those in older age and more deprived groups. A planned, concerted effort was made to boost ethnically diverse recruitment; however, most of the specific approaches to this end were implemented later in recruitment. High NNI values for the youngest, most-deprived, and Asian and Black individuals may reflect barriers related to competing demands on time, life stressors, mistrust in clinical trials and the healthcare system, perceived low personal relevance, and lower (health) literacy levels. Overall, our study used a range of methods to achieve diverse and inclusive recruitment, which can be refined and built upon by future clinical trials.

## Supplemental Material

sj-pdf-1-ctj-10.1177_17407745241302477 – Supplemental material for NHS-Galleri trial: Enriched enrolment approaches and sociodemographic characteristics of enrolled participants Supplemental material, sj-pdf-1-ctj-10.1177_17407745241302477 for NHS-Galleri trial: Enriched enrolment approaches and sociodemographic characteristics of enrolled participants by Charles Swanton, Velicia Bachtiar, Chris Mathews, Adam R Brentnall, Ian Lowenhoff, Jo Waller, Martine Bomb, Sean McPhail, Heather Pinches, Rebecca Smittenaar, Sara Hiom, Richard D Neal and Peter Sasieni in Clinical Trials
