# Supplementary material for: NHS-Galleri trial: Enriched enrolment approaches and sociodemographic characteristics of enrolled participants
Source: Clin Trials. 2025 Jan 25;22(2):227–38. doi: 10.1177/17407745241302477 (PMC11986080; doi:10.1177/17407745241302477)
Supplement: sj-pdf-1-ctj-10.1177_17407745241302477 – Supplemental material for NHS-Galleri trial: Enriched enrolment approaches and sociodemographic characteristics of enrolled participants [file sj-pdf-1-ctj-10.1177_17407745241302477.pdf]

Supplemental Material

Cancer Alliances participating in the NHS-Galleri trial.

All individuals invited to participate in the trial were registered at a general practice in one of eight Cancer Alliance regions in England at the time of invitation: Northern, Cheshire and Merseyside, Greater Manchester, West Midlands, East Midlands, East of England (North), South East London, and Kent and Medway. These eight Cancer Alliances were selected from a total of 21 Cancer Alliances across England based on their relatively high levels of socioeconomic deprivation and cancer mortality, and poor early-stage diagnosis.

Development of invitations, information sheets, and consent forms used in the NHS-Galleri trial.

Briefly, five members of the public each took part in two one-on-one, face-to-face, 90-minute, semi-structured interviews with behavioural researchers at King's College London to review and improve accessibility and comprehension of these materials. In addition, 32 individuals from patient and public involvement groups in four of the eight Cancer Alliances included in the trial gave feedback on the invitation and how they would feel to receive it. Focus groups involving over 50 individuals from more socioeconomically deprived neighbourhoods and a range of ethnic backgrounds were also held to understand attitudes towards the trial, potential barriers and motivations to taking part in the trial, and trial information requirements of invitees and participants. These groups were run by a specialist communications agency. Finally, the NHS DigiTrials team consulted a panel of 11 individuals representing both sexes and a range of ages, ethnicities, and regions in England to provide feedback on the trial invitation. This informed the language on opting out of the NHS-Galleri trial and clinical trials in general that was used in the invitation letter and on the trial website.

Accessibility measures used during appointments and calls to the call centre

Step-free access (n = 7727, 5.4%); language interpretation (n = 858, 0.6%) in 45 languages other than English; and visual and hearing assistance (n = 144, 0.1%).

List of languages requested for on demand interpretation at the point of registration and during appointments.

Albanian
Amharic
Arabic
Bengali
British Sign Language/Sign Language
Bulgarian
Cantonese
Croatian
Czech
Egyptian Arabic
Farsi
French
Greek
Gujarati
Hindi
Hungarian
Italian
Japanese
Kashmiri
Kurdish
Latvian
Lithuanian
Malayalam
Maltese
Mandarin
Nepali
Polish
Portuguese
Punjabi
Pushto; Pashto
Romanian
Russian
Serbian
Sinhala
Slovak
Somali
Spanish
Tamil
Thai
Tigrinya
Turkish
Twi (Ghanaian)
Ukrainian
Urdu
Vietnamese

Further detail on translated materials and language interpretation.

Translated materials were available in Bengali, Urdu, Gujarati, and Punjabi because these were the four most commonly spoken languages among minority ethnic groups in England according to the most recent census (2011) for which data were available when this decision was made. Other factors were also considered, such as whether native speakers of languages other than English would be likely to speak English as a second language, have access to family members that could translate, or use any translated materials.

Two types of language interpretation services were made available to participants to remove barriers to taking part. The first type was on-demand language interpretation, which was available through the call centre and enabled invitees to register to take part and book an appointment. The second was language interpretation available during the appointment via a video link to support participants through the clinic visit and consent process. An external third-party vendor certified in medical language interpretation was used.

England and Cancer Alliance Comparator Data

For the total population of equivalent age across the Cancer Alliances, we used Office for National Statistics (ONS) 2021 census data for age, sex, ethnicity, and IMD.[29–32] The age range was 50–77 years for age, sex, and IMD, and ≥50 years for ethnicity. The 2021 census population by IMD quintile was constructed using LSOA-level population estimates by sex and single year of age, linked to LSOA-level IMD data.[26]

For the total population aged 50–77 years in England, we used the most recently available England data that matched this age range as closely as possible: ONS 2021 census data for those aged 50–77 years for age and sex[29,30]; ONS 2021 population estimates for those aged ≥50 years for ethnicity[31]; ONS 2021 population estimates by IMD decile for those aged 50–77 years for IMD, constructed as described for the Cancer Alliance population[32]; and NHS Digital 2019 Health Survey for England data for those aged 45–54, 55–64, 65–74, and ≥75 years for BMI, smoker status, and alcohol drinker status.[33]

Definitions of smoker and alcohol drinker categories used in this study.

In the NHS-Galleri trial participant survey, those who reported ‘currently smoking cigarettes regularly’ were defined as ‘current smokers’; those who were not current smokers but who had smoked at least 100 cigarettes in their lifetime were ‘former smokers’, and those who had smoked less than 100 cigarettes in their lifetime were ‘non-smokers’. In the Health Survey for England, those who reported ‘smoking at all nowadays’ were defined as ‘current smokers’, those who had ever smoked at all were ‘former smokers’, and those who had never smoked at all were ‘non-smokers’.

In the NHS-Galleri trial participant survey, those who reported ‘currently drinking alcohol’ were defined as ‘current drinkers’; those who were not current drinkers but who had consumed any alcohol in the past were defined as ‘former drinkers’; and those who had never consumed any alcohol were ‘non-drinkers’. In the Health Survey for England, those who reported ‘ever drinking alcohol in the past year’ were defined as ‘current drinkers’; those who had never consumed alcohol and those who had last consumed alcohol over a year ago were combined into one single category, ‘former and non-drinkers’.

Table S1. Demographic characteristics of the participants enrolled in the NHS-Galleri trial compared with the England and Cancer Alliance populations according to 2011 census data. produced by the Office for National Statistics (available at: https://www.ons.gov.uk/census/2011census, accessed 16 August 2023). Data are presented as n (%). Percentages may not sum to 100% across groups due to rounding error.

		NHS-Galleri Trial	Cancer Alliance Regions[a]	England
Years of Age[b]	50–54	15,436 **(10.8)**	1,562,000 **(22.1)**	3,400,000 **(22.4)**
	55–59	22,460 **(15.7)**	1,391,000 **(19.7)**	3,000,000 **(19.8)**
	60–64	26,815 **(18.8)**	1,484,000 **(21.0)**	3,172,000 **(20.9)**
	65–69	30,813 **(21.6)**	1,180,000 **(16.7)**	2,508,000 **(16.5)**
	70–74	30,428 **(21.3)**	963,000 **(13.6)**	2,044,000 **(13.5)**
	75–77	16,631 **(11.6)**	487,000 **(6.9)**	1,044,000 **(6.9)**
Sex[b]	Female	71848 **(50.3)**	3,777,000 **(51.3)**	7,775,000 **(51.3)**
	Male	71076 **(49.7)**	3,580,000 **(48.7)**	7,392,000 **(48.7)**
Ethnicity[c]	White	133,171 **(93.2)**	8,025,000 **(94.8)**	14,663,000 **(92.9)**
	Asian	4725 **(3.3)**	259,000 **(3.1)**	669,000 **(4.2)**

		NHS-Galleri Trial	Cancer Alliance Regions[a]	England
	**Black**	2056 **(1.4)**	120,000 **(1.4)**	289,000 **(1.8)**
	**Other**	627 **(0.4)**	24,000 **(0.3)**	79,000 **(0.5)**
	**Mixed**	1544 **(1.1)**	39,000 **(0.5)**	91,000 **(0.6)**
**IMD Quintile[b,d]**	**1 - Most Deprived**	32,347 **(22.7)**	1,495,000 **(21.2)**	2,496,000 **(16.5)**
	**2**	28,067 **(19.6)**	1,335,000 **(18.9)**	2,773,000 **(18.3)**
	**3**	30,125 **(21.1)**	1,439,050 **(20.4)**	3,150,000 **(20.8)**
	**4**	28,963 **(20.3)**	1,454,398 **(20.6)**	3,341,000 **(22.0)**
	**5 - Least Deprived**	22,953 **(16.0)**	1,343,337 **(19.0)**	3,406,000 **(22.5)**

[a]Cancer Alliances were: Northern, Cheshire and Merseyside, Greater Manchester, West Midlands, East Midlands, East of England (North), South East London, and Kent and Medway.

[b]For age, sex and IMD, the age range for Cancer Alliance and England data was 50–77 years.

[c]For ethnicity, the age range for Cancer Alliance and England data was 50–79 years.

[d]IMD quintile was based on LSOAs in England.

IMD: index of multiple deprivation; LSOA: lower-layer super output area.

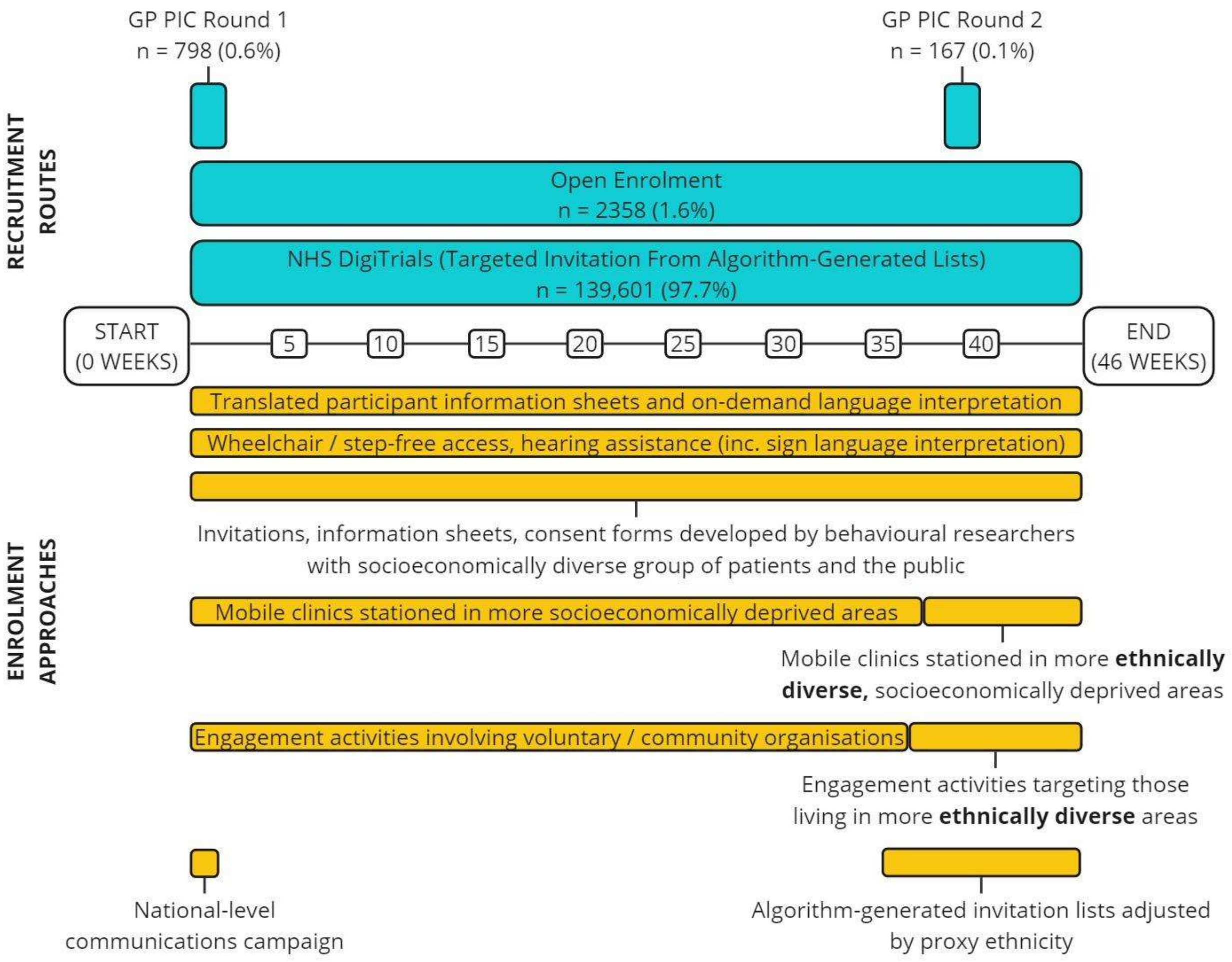

Figure S1. Recruitment routes and enrolment approaches used in the NHS-Galleri trial. The number of participants recruited via each route (also expressed as a percentage of the total number recruited in the trial) are provided. GP PIC: general practice participant identification centre.

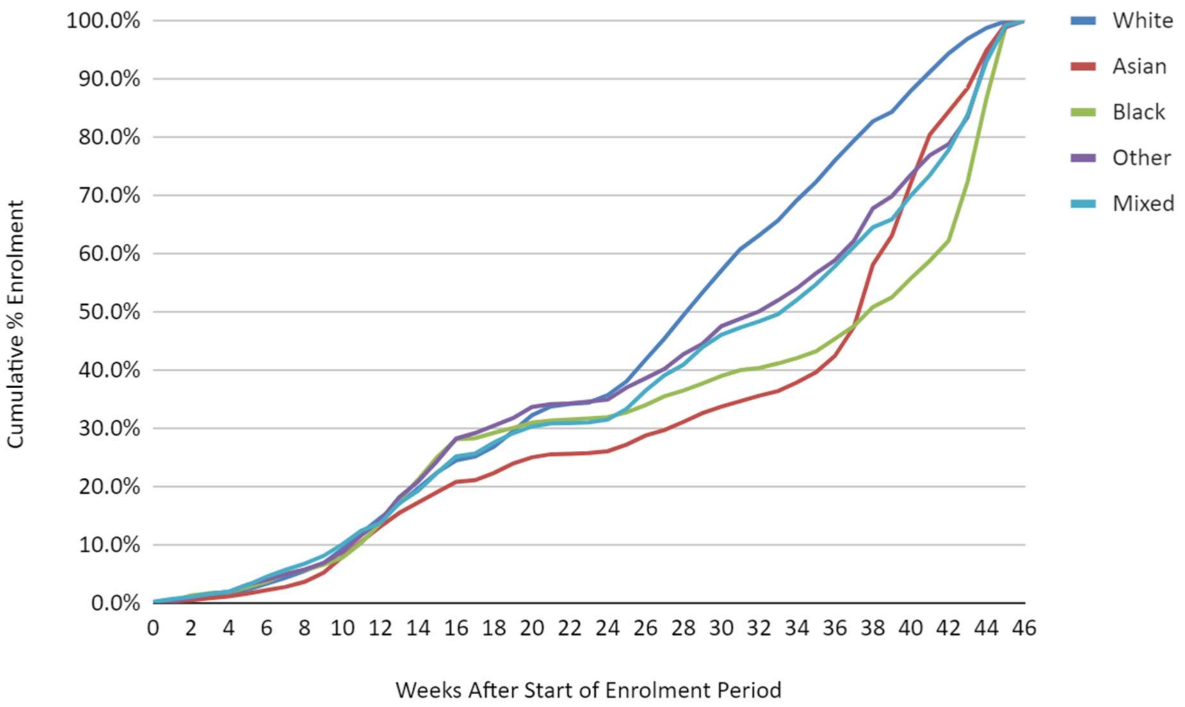

Figure S2. Weekly cumulative percentage of participants enrolled in the NHS-Galleri trial throughout the 10.5-month enrolment period (31 August 2021 to 26 July 2022) by ethnicity. The denominator for each ethnicity group corresponds to the total number enrolled in that specific ethnicity group.
